# Risk Perception and Preparedness of Undergraduate Dental Students to Treat Patients in View of COVID-19 Pandemic: A Questionnaire Survey

**DOI:** 10.1155/2022/4489773

**Published:** 2022-12-21

**Authors:** Anuprita Nair, Nishu Singla, Ritesh Singla, Arhana De

**Affiliations:** ^1^Manipal College of Dental Sciences, Manipal, Manipal Academy of Higher Education, Manipal 576104, Karnataka, India; ^2^Department of Public Health Dentistry, Manipal College of Dental Sciences, Manipal, Manipal Academy of Higher Education, Manipal 576104, Karnataka, India; ^3^Department of Orthodontics & Dentofacial Orthopaedics, Manipal College of Dental Sciences, Manipal, Manipal Academy of Higher Education, Manipal 567104, Karnataka, India

## Abstract

With the gradual resumption of dental services worldwide, it is crucial to focus on returning dental undergraduates to their clinical postings. The assessment of foreseeable concerns from a student's point of view will help the dental schools tailor a comprehensive plan of action that would be in the best interest of everyone. *Aim*. Hence, this survey was planned to assess dental undergraduates' risk perception and preparedness to provide patient care amidst the COVID-19 pandemic crisis. *Material and Methods*. It was an online survey carried out among students involved in clinical work at two dental colleges in Manipal and Mangalore, respectively, in Karnataka, India. The online questionnaire was sent to approximately 500 students, with responses from 301 students. The survey comprised 21 closed-ended questions about demographics, risk perception, and preparedness. The descriptive statistics were done on the data. *Results*. It was found that all the students (99.7%) perceived COVID-19 to be dangerous, and 73.4% chose to avoid treating those patients suspected to have an active COVID-19 infection. The fear of being infected was perceived by 55.1% of students, while 46.2% feared transmitting the infection to friends and family. A majority (87.7%) believed standard infection controls practiced prior to the pandemic were insufficient to work in the current scenario. Nearly 33.6% could not view the guidelines for dental procedures during the pandemic. A majority (87.7%) were not/little confident, and 61.7% were unsure/unprepared to manage suspected patients. *Conclusion*. It is the prime need of the hour for dental schools to instill self-reliance within students in managing patient care under these circumstances by strictly reinforcing the official protective care guidelines.

## 1. Introduction

A significant part of the world halted on the 11th of March 2020 when the World Health Organization (WHO) confirmed the SARS-CoV-2 virus outbreak in China's Hubei province as a global pandemic. From government-imposed lockdowns and canceled flights to the closure of public spaces of all kinds, every effort was made to curb the spread of the Coronavirus. Dental operations are known to be the “highest risk” of the possible spread of various respiratory viruses, as reported by the US Centers for Disease Control (CDC) and the Occupational Safety and Health Administration [1]. To avoid spreading of Coronavirus disease (COVID-19 infection), in March 2020, dental colleges worldwide shut their teaching clinics and switched to an online mode of conducting classes and examinations after the implementation of lockdown measures. However, dental hospitals stayed open to ensure essential access to immediate nonhospital-based urgent care for citizens in dental emergencies. Faculty clinicians and postgraduate students provided these emergency services [2].

From a theoretical perspective, during the COVID‐19 crisis, various academic activities have transitioned to an online mode of instruction [2]. The dental faculty constructed an online curriculum comprising virtual lectures and group discussions to ensure the continuity of learning. They utilized the latest technologies such as video teleconferencing and file-sharing platforms to enhance student participation. However, despite best efforts, the students lost opportunities to develop and work on their clinical skills, an integral part of dental education. The preplanned schedule of preclinical activities, clinical rotations, and examinations was significantly disturbed.

Moreover, by stopping patient care abruptly, there were raising concerns of academic institutions ensuring clinical competency of students [2]. It was difficult to adhere to guidelines and fulfil the necessary requirements of dental curriculum enforced by the licensing bodies such as Dental Council of India (DCI). Due to the technical difficulties and social risks associated with conducting entrance examinations for the new incoming dental batches, the intended schedule for the commencement of a new dental student class was also disturbed significantly. It had created a hurdle in the flow of preclinical and clinical training of current dental students across the country.

COVID-19 remains a significant issue for global health, economy, and society although strict lockdown measures are being relaxed at most of the places. Resuming institutional and educational activities is still hampered due to the repeated surges of COVID-19 cases and the emergence of new virus variants [3]. It is ought to abide by the various guidelines released by international and national dental regulatory bodies for managing dental patients [1]. The challenges identified concerning the same include protecting the health of dental care providers, ensuring the continuation of dental education, ensuring the safety of procedures and adhering to guidelines. Due to the fear and uncertainty about such situations, it is necessary to have excellent communication of roles, duties, policies, and support structures within dental schools.

Much research has been done on the consequences of the Coronavirus disease pandemic to dental training and education since its onset. However, as we move forward and start preparing for this change towards a subnormal period, we must consider the associated physical risk and mental toll of these changes on students and make sure that this transitory phase is implemented smoothly. Therefore, being well informed about the students' risk perceptions, knowledge, attitudes, and preparedness is necessary. Hence, this survey was planned to assess dental undergraduates' risk perception and preparedness to provide patient care amidst the COVID-19 pandemic crisis.

## 2. Material and Methods

An online survey was carried out among students involved in the clinical work at two dental colleges in Manipal and Mangalore, respectively, in Karnataka, India. Before the initiation of the survey, permission from the Institutional Ethics Committee was attained along with informed consent from all the participants (IEC: 805/2020). Additionally, the study was registered in the Clinical Trials Registry-India (CTRI) before enrolment of the first participant (Reg No. CTRI/2021/01/030647). The survey included a participation information sheet, and informed consent was obtained prior to accessing the questionnaire. Also, the anonymity of participants was maintained throughout the study.

The maximum sample size to carry out the study was considered as about 384 students by taking 50% expected proportion (*p*) at 95% CI with a 5% margin of error. We considered a high nonresponse rate of online questionnaires; hence, approximately 500 hundred students were reached by sending the questionnaire through their email ids. Participants involved in the clinical work as per the course curriculum (i.e., third years, fourth years, and interns) and willing to answer the questionnaire with informed consent were included. Candidates whose work is mainly limited to the preclinical section (i.e., first and second years) and candidates who did not respond to the questionnaire were excluded from the study.

The questionnaire was designed by taking guidance from two questionnaires from previously published similar articles [4, 5]. The permission letters for these have been obtained from the respective authors. Few questions were modified as per the need of our study. The English language of the questions was not changed. The survey comprised 21 closed-ended questions, which took about 5–7 minutes to complete. The questionnaire consisted of questions about demographics, risk perception, and preparedness. The face validity as well as the content validity of the questions were checked by the four subject experts [6]. The data were evaluated by descriptive statistics with SPSS Inc., Chicago, IL, USA software version 20.0.

## 3. Results

The questionnaire survey recorded responses from 301 respondents which included 209 females (69.4%) and 92 males (30.6%) with age between 20 and 26 years and a mean age of 22.3 ± 1.2 years. The study population was a mix of dental students in the 3^rd^ year (39.5%, *n* = 119), 4^th^ year (27.6%, *n* = 83), and intern year (32.9%, *n* = 99) of their education (Table 1).

Table 2 demonstrates the risk perception of the study population. It was found that all the students (99.7%, *n* = 300) perceived COVID-19 to be dangerous, and 73.4% (*n* = 221) chose to avoid treating patients suspected to have an active COVID-19 infection. The fear of being infected was perceived by 55.1% of students, while 46.2% feared transmitting the infection to friends and family. A majority (87.7%) believed that standard infection controls practiced prior to the pandemic were insufficient to work in the current scenario. The greatest risk of accidental exposure to COVID-19 during clinical postings was discerned in the Department of Conservative Dentistry and Endodontics by 49.5% students (*n* = 149). In addition, 9.3% students (*n* = 28) were hesitant to treat recovered COVID-19 patients.

**Figure 1:**
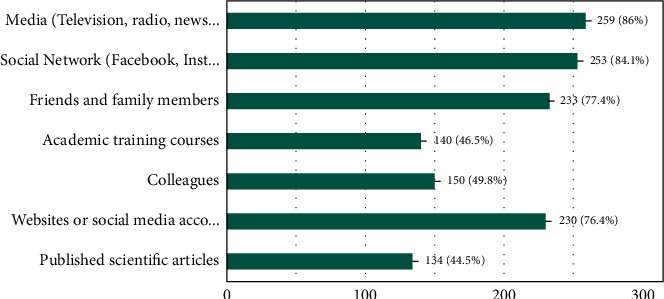
What are your sources of information about the COVID-19? (Select all that apply) 301 responses.

Figure 1 displays that majority of the participants reported media 86% (*n* = 259) and social media 84.1% (*n* = 253) to be their major sources of information regarding COVID-19. Other notable sources included friends and family members (77.4%, *n* = 233), and professional organizations such as WHO, CDC, Ministry of Health and Family Welfare, Government of India (MOHFW) etc. (76.4%, *n* = 230). Less common sources of information were published scientific articles (44.5%, *n* = 134), academic training courses (46.5%, *n* = 140), and colleagues (49.8%, *n* = 150).

Table 3 shows the preparedness of the study population for handling the patients. Nearly 33.6% (*n* = 101) could not view the national guidelines for dental procedures during the pandemic. A majority (87.7%, *n* = 262) were not/little confident and 61.7% were unsure/unprepared to manage suspected patients. A total of 11% (*n* = 33) respondents did not even know whom to approach in case of inadvertent exposure. In response to the changes in standard infection control measures after the COVID-19 pandemic ended, 79.1% respondents (*n* = 238) believed that they will be more careful.

**Figure 2:**
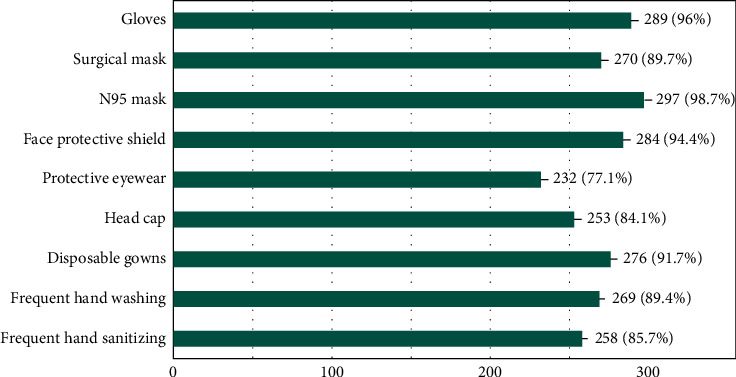
Availability and use of following measures will alleviate my concerns about treating patients in given times? (select all that apply) 301 responses.

**Figure 3:**
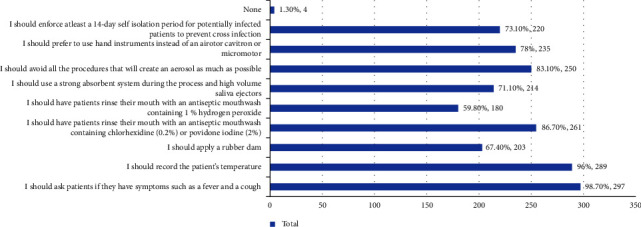
Precautions that you should be taking for the patients regarding COVID-19 while performing dental procedures. (select all that apply) 301 responses.

Figure 2 displays that the availability of gloves, surgical masks, N95 masks, disposable gowns, face protective shield, protective eyewear, and head caps was found to alleviate the concerns. Responses for precautions to be taken while treating patients are shown in Figure 3. It was found that hygiene maintenance by frequent hand washing and hand sanitizing also significantly contribute to alleviate the concerns about treating patients in given times.

## 4. Discussion

The dental education is greatly influenced by the COVID-19 pandemic. The likelihood of transmission of the deadly virus to the dental practitioners, the nurses, and other supporting staff in the dental setup is too high [7]. According to the study findings, most of the students chose to avoid treating COVID-19 suspects and perceived it to be very dangerous. The students were not found confident enough in treating patients and were unsure of their abilities and preparedness to manage patients in times of COVID-19. It justifies the need for specific crisis management training within the dental curriculum during health emergencies such as epidemics, pandemics, and natural disasters. This should include how to obtain reliable information, maintain proper communication with patients, and provide emergency dental care during a crisis [8].

It is crucial to increase awareness about dental treatment guidelines or other infection control protocols during the pandemic among the students to ensure a safe working environment at the dental hospital. In the present study, only 2/3rd of the study respondents had reviewed the ICMR/DCI protocol for dental treatments during the pandemic [9]. However, it was noticed that half of the study respondents were not familiarized with the correct set of guidelines. Failure to do so can put the dentists and other dental staff along with the patients with increased chances of exposure to COVID-19 virus. This study highlights that majority of study participants perceived that standard infection control measures used prior to onset of the COVID-19 pandemic would not suffice to treat patients safely in these times.

The most significant risk of exposure was believed by the students to be in the Conservative Dentistry and Endodontics Department followed by Periodontology Department as it reflects upon the student's ability to perceive the risk due to the knowledge about the aerosol generation associated with air rotor devices and ultrasonic scalers [5]. Notably, the study found that no risk was perceived by respondents in performing treatments in the Pediatric Dentistry and Orthodontics Department. However, as per the latest report from the Ministry Of Health and Family Welfare (MOHFW) outlining operational care guidelines for COVID services for children and adolescents, experts are expecting a future surge of cases with a maximum threat among this population [10]. Therefore, there is a need to prepare for any sudden rise of COVID cases in this age group, and it is essential to educate them.

The management guidelines of recovered COVID-19 patients also need to be clarified among students to encourage confidence and prepare students in treating such patients. The study found that 36.2% were unsure about treating recovered COVID-19 patients. According to the CDC guidance for dental settings, it is safe to perform dental treatment for people who have completed their home quarantine period [11]. However, another study finds the reactivation of COVID-19 following 14 days, thus raising the question if the specified time is sufficient or if the dentist should defer dental treatment for up to 28 days [12]. Also, it is crucial to carefully evaluate the oral tissues for signs of COVID-19 associated Mucormycosis, especially in patients who have severe COVID-19 infection history and diabetes mellitus [13].

The major source of information regarding the pandemic were reported to be the mass communication sources. The study highlights that although social media is an influential, widely accessible tool for filling voids regarding crucial details, it can also quickly accelerate the spread of incorrect, harmful information, bringing out health behavior changes, resulting in an “Infodemic” as reported by WHO [5]. Notably, less than half of the respondents relied on academic training courses and published scientific studies for information [14]. It is essential to encourage scientific published pieces of evidence to avoid misinformation, especially among dental students. It is the duty of dental institutions to organize more webinars and virtual conferences to reiterate and update students and faculty regarding the most recent COVID-19 management guidelines.

The majority of participants feared that they could transmit COVID-19 to their friends and family members due to the nature of their profession, thus highlighting the need for more stringent measures of protection. Employing minimal invasive procedures, avoiding use of air syringe and opting for atraumatic restorative treatment options, when possible, will reduce the risk associated with aerosols [15]. Applying such measures will equip students and faculty to successfully practice in challenging circumstances and confidently face any future disease epidemics and pandemics. The availability and use of N95 masks would alleviate the concerns of almost 99% of respondents. The provision of the personal protective devices is the responsibility of the dental colleges to ensure ease and safety in functioning for their students, faculty, and auxiliaries. Strict compliance to frequent handwashing and sanitizing practices as instructed by WHO would further curb the fears associated with working in a high-risk dental setup. Additionally, educating the dental students about donning and removing personal protective equipment (PPE) attire before and after the treatment is necessary.

Majority of the students emphasized on obtaining a thorough history of symptoms such as fever, cough, and recording patient temperature and also some of the specific measures they would follow to protect themselves and their patients during dental treatments. If any symptomatic patients are found, they should visit a hospital at the earliest and undergo a mandatory 14-day self-isolation before seeking dental care [9]. This highlights the importance of prescreening and triage before the commencement of procedures. It is best to postpone nonemergent, elective, aerosol-generating procedures to a later date if possible. DCI guidelines encourage the use of preprocedural mouth rinse with povidone (0.2%) or hydrogen peroxide (1.5%). Additional perioperative measures like a robust absorbent system, high volume ejectors, rubber dam, and hand instruments over the air rotor, cavitron, and micromotor will help reduce risk to the patient and practitioner. A 3-in-1 syringe creates droplets due to the forcible ejection of water/air; hence, its usage must be avoided/minimized [9].

Self-protection is of utmost importance; 89.4% of respondents knew who to approach if there is an accidental exposure to a confirmed or COVID-19 suspects. 99% of them were aware of what procedures to follow if they acquire any symptoms of this infection. It reflects on the students' awareness to respond to such a situation. Timely reporting, immediate testing, notifying authorities about contact history, and self-isolating are essential to prevent the further spread of infection within the community.

Assessment of the student risk perception and preparedness is crucial in ensuring a safe working and learning environment for dental students. It will train them to combat high stress and high-risk situations like these and effectively manage their dental practices and ensure safety and protection for their patients as well in any future epidemics/pandemics. Having well trained, confident, and assured dental teams will also help ensure that the patient's oral health is not neglected and timely treatment is provided under safe conditions [16].

### 4.1. Limitations

This study has a small sample size comprising of dental students in two dental colleges only. The infection control measures may or may not meet the recommended standards set by Dental Council of India at all times in various dental schools. This variability can cause students to perceive the risk of COVID-19 differently and be more or less prepared to manage patients amidst the COVID-19 pandemic.

## 5. Conclusion

With gradual resumption of dental services across the world, it is important to focus on training of dental students to such situations. From the provision of adequate training via lectures and demonstrations on the enhanced infection control protocols being followed in the clinics to the availability and strict use of personal protective equipment will help alleviate some of these concerns. It is the prime need of the hour for dental schools, universities, and hospitals to instill self-reliance within dental students in managing patient care under these circumstances by reinforcing the recommended protective care guidelines by the DCI, ICMR, and MOHFW through seminars and workshops. Addressing common myths and misconceptions and providing abundant, good quality, personal protective equipment will help achieve increased self-reliance. Most importantly, encouraging the spread of accurate scientific information regarding COVID-19 and educating their family, friends, and patients will help them be well-informed representatives of the healthcare workforce. Assessment of foreseeable concerns from a student's point of view will help the respective dental schools tailor a comprehensive plan of action that would be in the best interest of everyone.

## Figures and Tables

**Table 1:** Demographics of the study population.

Variable	Range	Mean ± SD
Age	20–26 years	22.3 ± 1.2

		*N* (%)
Gender	Female	209 (69.4%)
	Male	92 (30.6%)

Class	3^rd^ year	119 (39.5%)
	4^th^ year	83 (27.6%)
	Interns	99 (32.9%)

	Total	301 (100%)

**Table 2:** Risk perception of the study population.

Variable		*N* (%)
(1) How do you perceive COVID-19?	Very dangerous	210 (69.8%)
	Moderately dangerous	90 (29.9%)
	Not dangerous	1 (0.3%)

(2) COVID-19 symptoms often resolve with time and do not require any special treatment.	Yes	181 (60.1%)
	No	120 (39.9%)

(3) Do you prefer to avoid working with a patient who is a suspect of COVID-19?	Yes	221 (73.4%)
	No	80 (26.6%)

(4) In case a patient was sneezing or coughing in your clinic, what would you do?	Refuse treating the patient and ask him/her to leave the clinic	22 (7.3%)
	Treat the patient and ask him/her to go to the hospital	121 (40.2%)
	Refer the patient to the hospital without treating him/her	158 (52.5%)

(5) Are you afraid of becoming infected with COVID-19 as a healthcare professional working at close range with the patient?	Yes	166 (55.1%)
	No	37 (12.3%)
	Not sure	98 (32.6%)

(6) Do you fear that you will transmit COVID-19 to friends and family members?	Strongly agree	139 (46.2%)
	Agree	154 (51.2%)
	Disagree	8 (2.7%)
	Strongly disagree	0

(7) Do you think, it is not safe to work with the standard precautions which were followed before the onset of COVID-19 pandemic?	Strongly agree	119 (39.5%)
	Agree	145 (48.2%)
	Disagree	32 (10.6%)
	Strongly disagree	5 (1.7%)

(8) Do you feel the crisis of COVID-19 pandemic increased your workload?	Strongly agree	93 (30.9%)
	Agree	119 (39.5%)
	Disagree	69 (22.9%)
	Strongly disagree	20 (6.6%)

(9) In which clinical department posting are you most concerned about accidental exposure to COVID-19 during treatment?	Oral medicine and radiology	12 (4%)
	Conservative dentistry and endodontics	149 (49.5%)
	Periodontics	55 (20.1%)
	Oral and maxillofacial surgery	60 (19.9%)
	Prosthodontics	16 (5.3%)
	Pedodontics	0
	Orthodontics	0
	Public health dentistry	5 (1.7%)

(10) Would you hesitate to treat a patient who came for dental treatment after recovering from COVID-19 infection?	Yes	28 (9.3%)
	No	168 (55.8%)
	Not sure	105 (34.9%)

	Total	301 (100%)

**Table 3:** Preparedness of the study population.

Variable		*N* (%)
(11) Are you up to date on the latest information about case definitions for COVID-19?	Yes	260 (86.4%)
	No	41 (13.6%)

(12) Did you have the opportunity to look at the ICMR/Dental Council of India protocol for dental treatment procedures due to COVID-19 pandemic?	Yes	200 (66.4%)
	No	101 (33.6%)

(13) To what extent do you have confidence in handling suspected COVID-19 patients?	Not confident at all	40 (13.3%)
	Confident to a little extent	120 (39.9%)
	Confident to some extent	102 (33.9%)
	Confident to a considerable extent	31 (10.3%)
	Confident to a great extent	8 (2.7%)

(14) Do you consider yourself prepared for the management of patients during the COVID-19 outbreak?	Yes	115 (38.2%)
	No	48 (15.9%)
	Maybe	138 (45.8%)

(15) Do you believe that asking patients to set far from each other, wearing masks while in the waiting room, and washing hands before getting in the dental chair is?	Necessary and help to decrease disease transmission	298 (99%)
	Not necessary and could cause panic	3 (1%)

(16) Do you know whom to contact in a situation where there has been an unprotected exposure to a known or suspected COVID-19 patient?	Yes	268 (89%)
	No	33 (11%)

(17) Do you know what to do if you have signs or symptoms suspected of COVID-19 infection?	Yes	298 (99%)
	No	3 (1%)

(18) Do you think that after your COVID-19 pandemic, you will be more careful in your standard infection control measures regarding cross infection in your patients?	Yes	238 (79.1%)
	No	1 (0.3%)
	Undecided	62 (20.6%)

	Total	301 (100%)

## Data Availability

The study data set is available on request from Ritesh Singla (ritesh.singla@manipal.edu).
